# Single cell analysis reveals satellite cell heterogeneity for proinflammatory chemokine expression

**DOI:** 10.3389/fcell.2023.1084068

**Published:** 2023-03-27

**Authors:** Alexander B. Andre, Katherina P. Rees, Samantha O’Connor, Grant W. Severson, Jason M. Newbern, Jeanne Wilson-Rawls, Christopher L. Plaisier, Alan Rawls

**Affiliations:** ^1^ School of Life Sciences, Arizona State University, Tempe, AZ, United States; ^2^ Molecular and Cellular Biology Graduate Program, Arizona State University, Tempe, AZ, United States; ^3^ School of Biological and Health Systems Engineering, Arizona State University, Tempe, AZ, United States; ^4^ Biomedical Engineering Graduate Program, Arizona State University, Tempe, AZ, United States

**Keywords:** satellite cells, chemokines, scRNA-seq, interferon, inflammation, muscle repair

## Abstract

**Background:** The expression of proinflammatory signals at the site of muscle injury are essential for efficient tissue repair and their dysregulation can lead to inflammatory myopathies. Macrophages, neutrophils, and fibroadipogenic progenitor cells residing in the muscle are significant sources of proinflammatory cytokines and chemokines. However, the inducibility of the myogenic satellite cell population and their contribution to proinflammatory signaling is less understood.

**Methods:** Mouse satellite cells were isolated and exposed to lipopolysaccharide (LPS) to mimic sterile skeletal muscle injury and changes in the expression of proinflammatory genes was examined by RT-qPCR and single cell RNA sequencing. Expression patterns were validated in skeletal muscle injured with cardiotoxin by RT-qPCR and immunofluorescence.

**Results:** Satellite cells in culture were able to express *Tnfa*, *Ccl2*, and *Il6*, within 2 h of treatment with LPS. Single cell RNA-Seq revealed seven cell clusters representing the continuum from activation to differentiation. LPS treatment led to a heterogeneous pattern of induction of C-C and C-X-C chemokines (e.g., *Ccl2*, *Ccl5*, and *Cxcl0*) and cytokines (e.g., *Tgfb1*, *Bmp2*, *Il18*, and *Il33*) associated with innate immune cell recruitment and satellite cell proliferation. One cell cluster was enriched for expression of the antiviral interferon pathway genes under control conditions and LPS treatment. Activation of this pathway in satellite cells was also detectable at the site of cardiotoxin induced muscle injury.

**Conclusion:** These data demonstrate that satellite cells respond to inflammatory signals and secrete chemokines and cytokines. Further, we identified a previously unrecognized subset of satellite cells that may act as sensors for muscle infection or injury using the antiviral interferon pathway.

## 1 Introduction

Skeletal muscle has the intrinsic capacity to repair itself in response to acute injury through crosstalk between the muscle and the innate immune system (reviewed in Tidball, 2017; Howard et al., 2020). Inflammatory signals, such as cytokines and chemokines, expressed at the site of injury, serve to activate and recruit monocytes and neutrophils responsible for phagocytosis of debris and necrotic fibers. These signals also promote a promyogenic signaling cascade directing the activation, proliferation, and differentiation of satellite cells (MuSCs), which are necessary to regenerate muscle fibers. Inhibition, or prolonged expression, of these factors diminishes muscle regeneration and promotes fibrosis (Dogra et al., 2006; Dogra et al., 2007; Munoz-Canoves et al., 2013). Despite the importance of cytokines and chemokines to this process, the contribution of muscle cells to immune cell recruitment and the proinflammatory environment remains poorly understood.

In response to acute injury, a proinflammatory environment is established at the site of injury that is necessary for the clearance of necrotic muscle fibers and cell debris (Yang and Hu, 2018). This is initiated immediately after tissue damage by resident neutrophils and degranulating mast cells releasing proinflammatory cytokines, including tumor necrosis factor-α (TNF-α), interferon-γ (IFN-γ) and interleukin-1β (IL-1β) (Gordon and Galli, 1990; Fielding et al., 1993). Subsequently, tissue resident macrophages responding to damage-associated molecular patterns (DAMPs) secrete TNF-α, IFN-γ, IL-1β, and IL-6, as well as chemoattractants, such as C-X-C-chemokine ligand 1 (CXCL1) and C-C-chemokine ligand 2 (CCL2) (Brigitte et al., 2010; Venereau et al., 2012; Yang and Hu, 2018). Collectively, these signals promote the recruitment of additional neutrophils and proinflammatory (M1) macrophages that produce reactive oxygen species necessary for the degradation of the damaged muscle fibers (Collins and Grounds, 2001; Teixeira et al., 2003). Single-cell transcriptomic analyses of regenerating muscle have demonstrated that macrophages, neutrophils, and fibroadipogenic progenitor cells (FAPs) residing in the endomysial space of muscle are the primary source of proinflammatory signals (Oprescu et al., 2020; McKellar et al., 2021). The importance of these signals to successful muscle repair has been demonstrated in mice that were deficient for *Tnfα*, *Ccl2*, or their respective receptors (Collins and Grounds, 2001; Warren et al., 2002; Chen et al., 2005; Lu et al., 2011). These mutations resulted in reduced muscle inflammation, as measured by decreased infiltration of neutrophils and M1 macrophages after injury, concomitant with a decrease in the clearance of necrotic fibers and diminished muscle fiber regeneration.

Proinflammatory cytokines also promote muscle repair by direct regulation of the myogenic progenitor cell population. Quiescent satellite cells are positioned between the basement and plasma membrane of muscle (Chargé and Rudnicki, 2004). They are distinguished by *Pax7* expression, but not the myogenic transcription factors, *Myod1*, *Myf5*, or myogenin (*Myog*). Upon injury, MuSCs start expressing *Myod1* and *Myf5* and pass through a period of rapid cell population expansion. Daughter cells undergoing differentiation to contribute to muscle fiber repair decrease *Pax7* expression and increase expression of a third myogenic transcription factor, *Myog*. IL-6 and IFN-γ secreted at the site of injury contribute to the activation, proliferation, and migration of the MuSC population (Munoz-Canoves et al., 2013; Fu et al., 2015). TNF-α differentially regulates MuSCs based on its level of expression, at high levels it promotes proliferation and migration and inhibits differentiation. As the inflammation is resolved, TNF-α levels decrease, and MuSCs begin to differentiate and form new muscle fibers (Li, 2003; Torrente et al., 2003; Langen et al., 2004; Dogra et al., 2006; Fu et al., 2015). C-C and C-X-C chemokines have been implicated in the migration, proliferation, and differentiation of satellite cells during muscle repair (Griffin et al., 2010; Ge et al., 2013; Zhu et al., 2016; Hogan et al., 2018). Thus, proinflammatory cytokines tightly coordinate the activity of both myeloid and myogenic cell lineages during muscle repair.

Proinflammatory factors also contribute to idiopathic inflammatory myopathies, including dermatomyositis (DM) and polymyositis (PM) (Kuru et al., 2003). The affected muscle expresses proteins associated with the activation of the type I interferon pathway (Boehm et al., 1997; Greenberg et al., 2005; Salajegheh et al., 2010; Suárez-Calvet et al., 2014). These observations and several *in vitro* studies raise the possibility that skeletal muscle can also contribute to activation of the inflammatory response associated with healthy muscle repair in response to injury. For example, the well-studied inflammatory activator, bacterial lipopolysaccharide (LPS), induced C2C12 and L6 myoblast cell lines to express cytokines (Frost et al., 2003). LPS treatment is used to stimulate sterile inflammation in culture (Frost et al., 2002) by acting on Toll Like Receptor 4 (TLR4), a cellular membrane sensor of damage and infection that also triggers the caspase-11 non-canonical inflammasome (Yang et al., 2015; Cieslelska et al., 2021). LPS treatment induced *Tnfα, Il6, Ccl2*, and *Cxcl1* expression within 2 h in these cell lines (Frost et al., 2002; Frost et al., 2003; Bivona et al., 2021). Primary human MuSCs have also been shown to express a broad set of cytokines and chemokines associated with muscle repair, including *Tnfa*, *Il6*, *Ccl2*, and *Ccl5* when treated with TNF-α or IFN-γ (De Rossi et al., 2000).

In this study, we wanted to explore whether MuSCs contributed to the proinflammatory response during healthy muscle repair post-injury. We observed that primary mouse MuSC cultures were comprised of cells along the continuum from activation through differentiation and they upregulated cytokine expression in response to LPS treatment. Single cell RNA sequencing (scRNA-Seq) revealed that a broad set of cytokines and chemokines were differentially expressed in response to LPS. For example, *Ccl2* and *Cxcl1* were broadly expressed throughout the culture. In contrast, a small subset of MuSCs expressed *Ccl5* and genes belonging to the antiviral IFN pathway. These cells were identifiable in PBS treated control cells, suggesting this is a stable cell population. Further, CCL5 positive MuSCs were detectable in cardiotoxin (CTX) injured muscle suggesting that chemokine and cytokine expression by these cells has a distinct function in muscle repair.

## 2 Materials and methods

### 2.1 Animals

B6129SF1/J mice were purchased from Jax Labs (Bar Harbor, ME) and bred and housed in a vivarium at Arizona State University (ASU). These mice were kept on a 10 h light: 14 h dark schedule with *ad libitum* access to food and water. ASU is accredited by the Association for Assessment and Accreditation of Laboratory Animal Care (AALAC). All procedures were carried out in compliance with the ASU institutional animal care and use committee and AALAC under an approved research protocol.

### 2.2 Cell culture and satellite cell isolation

Primary muscle satellite cell cultures (MuSCs) were established from cells isolated from five 12 week old B6129SF1/J mice as previously described (Palade et al., 2019). Briefly, hind limb quadriceps femoris muscles were excised, trimmed of fat and connective tissue, and finely minced. The muscle tissue was digested with 1.25 mg of protease XIV (Sigma-Aldrich, St. Louis, MO) for 1 h at 37°C. The cell suspension was filtered, differentially centrifuged and pre-plated in DMEM (Corning, Corning NY), containing 2% donor horse serum (HS) (Atlanta Biologicals, Flowery Branch, GA), and 100 μg/mL Primocin (Invivogen, San Diego, CA). Fibroblasts were removed by three pre-plating rounds of 3 h. Satellite cells were grown in a humidified chamber at 37°C with 5% CO_2_, on Matrigel Matrix Basement Membrane (BD Biosciences, Bedford, MA) coated plates, in growth medium, Hams F-10 (Corning), containing 20% FBS (Atlanta Biologicals), 10 ng/mL bFGF (BD Biosciences) and 100 μg/mL Primocin (Invivogen).

### 2.3 LPS induction and quantitative RT-PCR (RT-QPCR)

MuSCs were seeded onto Matrigel (BD Biosciences) coated 6 well plates at a density of 1 × 10^5^ cells/well in growth medium. Cells were then treated with LPS (1 mg/mL; Sigma-Aldrich) or phosphate buffered saline (PBS) for 1, 2, 4, 6, or 8 h. Cells were lysed in TRIzol (ThermoFisher Scientific, Waltham, MA) for RNA isolation, per the manufacturer’s protocol, and 2 μg of total RNA was used to synthesize cDNA using SuperScript III reverse transcriptase (Invitrogen, Carlsbad, CA) and random hexamer primers.

Quantitative Real Time PCR (RT-qPCR) was carried out using SYBRgreen (Eurogentec, Fremont, CA) on an ABI 7900 HT thermocycler using a 384 well format in 10 μL reactions. All samples were normalized to the *Gapdh* transcript and relative gene expression was calculated using ΔΔCt analysis (Haimes and Kelley, 2010). Primer sequences are listed in Supplementary Table S1. All data are the result of triplicates from 3 biological samples.

### 2.4 Single cell RNA sequencing

MuSCs were treated with LPS as above for 2 h. MuSCs were then trypsinized (0.05% trypsin 0.53 M EDTA, Mediatech, Manassas VA) to create a single cell suspension. Cells were then counted on an automated cell counter and RNA library preparation was performed by the ASU Genomics Core facility. Samples were processed using a 10X Chromium single cell 3′ GEM, Library and Gel Bead kit V3 with a Chromium single cell B chip. The quality of each library was determined using Agilent TapeStation automated electrophoresis. Samples were sequenced at a read depth of ∼31,000 reads per cell (BioFrontiers Sequencing Core, University of Colorado, Boulder). The 10X Genomics CellRanger v6.0.1 was used to align to the *Mus musculus* reference genome mm10-2020-A (GRCm39), quantify, and provide basic quality control metrics for the scRNA-seq data. Data was then analyzed utilizing the Seurat R package. Cells with < 2000 reads or >80,000 reads were discarded as well as cells with < 0.9% or >20% mitochondrial genes. Clusters of cells with similar expression patters were identified using the ‘FindClusters’ function which used a shared nearest neighbor modularity optimization-based clustering approach.

### 2.5 Cardiotoxin injury of skeletal muscle

Acute muscle injury was generated in the quadriceps of 3 month old mice by intramuscular injection of 50 μL of 10 μM cardiotoxin (CTX) (Sigma-Aldrich) solution in DMSO (Garry et al., 2000). At 24 h post-injection, Evan’s blue dye (Sigma-Aldrich) was injected intraperitoneally at 1 mg/10 g body weight to allow for visualization of damaged cellular membranes. Mice were sacrificed at designated time points and tissue was harvested for analysis. The uninjured quadriceps muscle from the contralateral leg was also harvested for use as control tissue. Total RNA was isolated from muscle biopsies using TRIzol (ThermoFisher Scientific) as described in Majumdar et al. (2012).

### 2.6 Immunofluorescence

Mice (n = 3) were injected with CTX, quadriceps were harvested, and fixed in 4% paraformaldehyde in PBS overnight at 4°C. Tissue was cryopreserved in sucrose prior to embedding in Tissue-Tek OCT. Sections were then permeabilized and blocked in a buffer containing 0.1% Triton-X 100% and 5% normal donkey serum (NDS, Sigma-Aldrich) in PBS. Samples were then incubated in primary antibody solution containing antibodies recognizing a muscle marker, M-cadherin (Santa Cruz #sc-81471 1:250) and CCL5 or CCL2 (CCL5, R&D Systems AF478 1:300, CCL2 Novus Biologicals NBP 1-07035 1:500). Sections were then rinsed and incubated in secondary antibody solutions containing Alexa-Fluor conjugated antibodies against mouse (Thermofisher, A-21202, 1:1000), rabbit (Thermofisher, A-31573, 1:1000) or goat IgG (Thermofisher, A-21447, 1:1000) and DAPI (Sigma Aldrich 10,236,276,001, 1:1000) diluted in blocking solution. Tissue was rinsed in PBS and cover-slipped for microscopic analysis. Images were collected on a Zeiss LSM800 laser scanning confocal microscope and optimized for brightness and contrast. Blinded evaluators quantified M-cadherin positive (MCAD^+ve^) and MCAD^+ve^/CCL5^+ve^ cells in random 20X fields. Data were averaged for both CTX injured (n = 418 MCAD^+ve^ cells) and contralateral control quadriceps (n = 416 MCAD^+ve^ cells). Student’s t-test was used to determine statistical significance.

MuSCs treated with LPS (1 mg/mL) or PBS control for 2 h, were prepared for immunofluorescence (IF) by washing twice with cold PBS followed by fixation in 4% PFA for 20 min. Cells were then washed 3 times with PBS before antibody staining. Monoclonal mouse anti-MYOD (NB100-56511, Bio-techne Minneapolis, MN, 1:200) and polyclonal goat anti-CCL5 (AF478-SP, R&D Systems, 1:200) were used to detect cellular expression in culture. Anti-mouse-Alexa Fluor 488 (1:1000), anti-goat Alexa Fluor 647 (1:1000) and DAPI (1:1000) were then used to visualize MYOD, CCL5, and nuclei, respectively. Images were collected on a Zeiss LSM800 laser scanning confocal microscope at 20X magnification. Cells from 50 fields were then counted to determine the percent of total cells positive for these markers.

### 2.7 Statistical analysis

To assess the significance of the expression of cultured MuSCs post-treatment a one-way ANOVA was used. The ‘FindMarkers’ function in the Seurat package was used to determine the significance of the differential expression of marker genes from each cluster in the scRNA-Seq data. Marker genes were determined as having an adjusted *p*-value less than 0.05 and an average log 2 fold-change greater than 0.25.

## 3 Results

### 3.1 Treatment of MuSCs with LPS induces inflammatory cytokine expression

**FIGURE 1:**
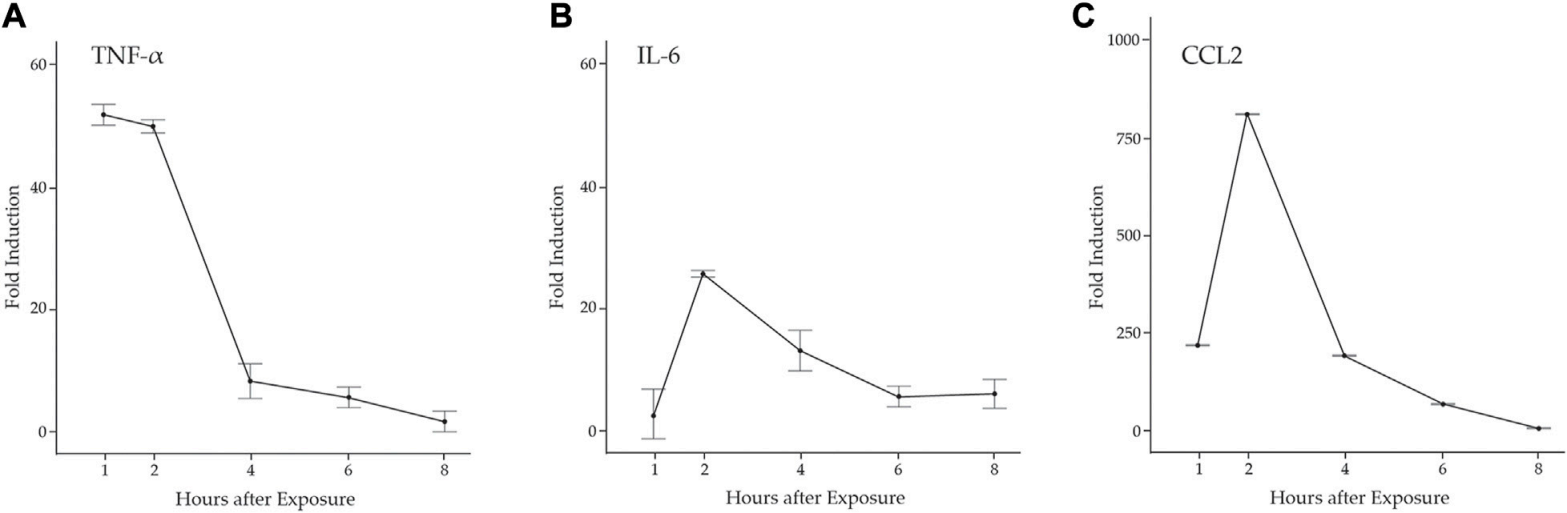
LPS induction of proinflammatory cytokines in primary mouse MuSCs. Transcription of **(A)** *Tnfα* **(B)** *Il6*, and **(C)** *Ccl2* from MuSCs treated with LPS (1 mg/mL) for 1, 2, 4, 6, and 8 h was quantified by RT-qPCR. RNA transcript levels are express as the fold induction over control MuSCs treated with PBS. At 1, 2, and 4 h, *p* < 0.001.

Proinflammatory cytokines promote neutrophil and M1 macrophage infiltration to the damaged muscle *via* diapedesis and promote the activation and proliferation of MuSCs necessary for muscle regeneration (Howard et al., 2020). There are several studies that indicate the potential for MuSCs to contribute to the proinflammatory environment. For example, the pathogen-associated molecular pattern molecule (PAMP) LPS induced the expression of *Tnfa*, *Il6, Ccl2, and Ccl5* in C2C12 and L6 muscle cell lines and human MuSCs (De Rossi et al., 2000; Frost et al., 2002; Frost et al., 2003). Further, expression of genes in the antiviral IFN pathway was elevated in the muscle of DM patients, predicting that muscle plays a role in the onset of inflammation (Moneta et al., 2019; Bivona et al., 2021). To examine the potential induction of an inflammatory response by MuSCs, we examined primary cell cultures derived from MuSCs isolated from the quadriceps of 3 month old B6129SF1/J mice treated with LPS (1 mg/mL) or a PBS control for 1, 2, 4, 6, or 8 h. Total RNA was isolated and transcription of *Tnfa, Il6*, and *Ccl2* was assessed by RT-qPCR. We found that *Tnfa* was rapidly induced and reached its maximum at 1 h post-treatment (*p* < 0.0001) with a significant drop off between 2 and 4 h. In contrast, *Il-6* and *Ccl2* transcription were induced to maximal levels at 2 h (*p* < 0.0001) (Figure 1).

### 3.2 Single cell RNA-SEQ analysis of primary MuSCs

It is becoming increasingly clear that MuSCs are heterogeneous based on the expression of specific markers, cell function, and their ability to contribute to muscle regeneration (Biressi and Rando, 2010; Rodriguez-Outeiriño et al., 2021). Thus, it is likely that the chemokine and cytokine profiles of individual MuSCs will vary across different subpopulations. To examine this, scRNA-Seq was performed on actively proliferating MuSCs treated with LPS or PBS. Based on the results in Figure 1, cells were exposed to LPS for 2 h for maximal induction of chemokines and cytokines. Single cells were isolated and profiled by scRNA-Seq. Following quality control for mitochondrial RNA content and reads per cell, this resulted in a PBS-treated library of 2,751 cells with 20,235 mean reads/cell and an LPS-treated library of 2,802 cells with 19,330 mean reads/cell.

**FIGURE 2:**
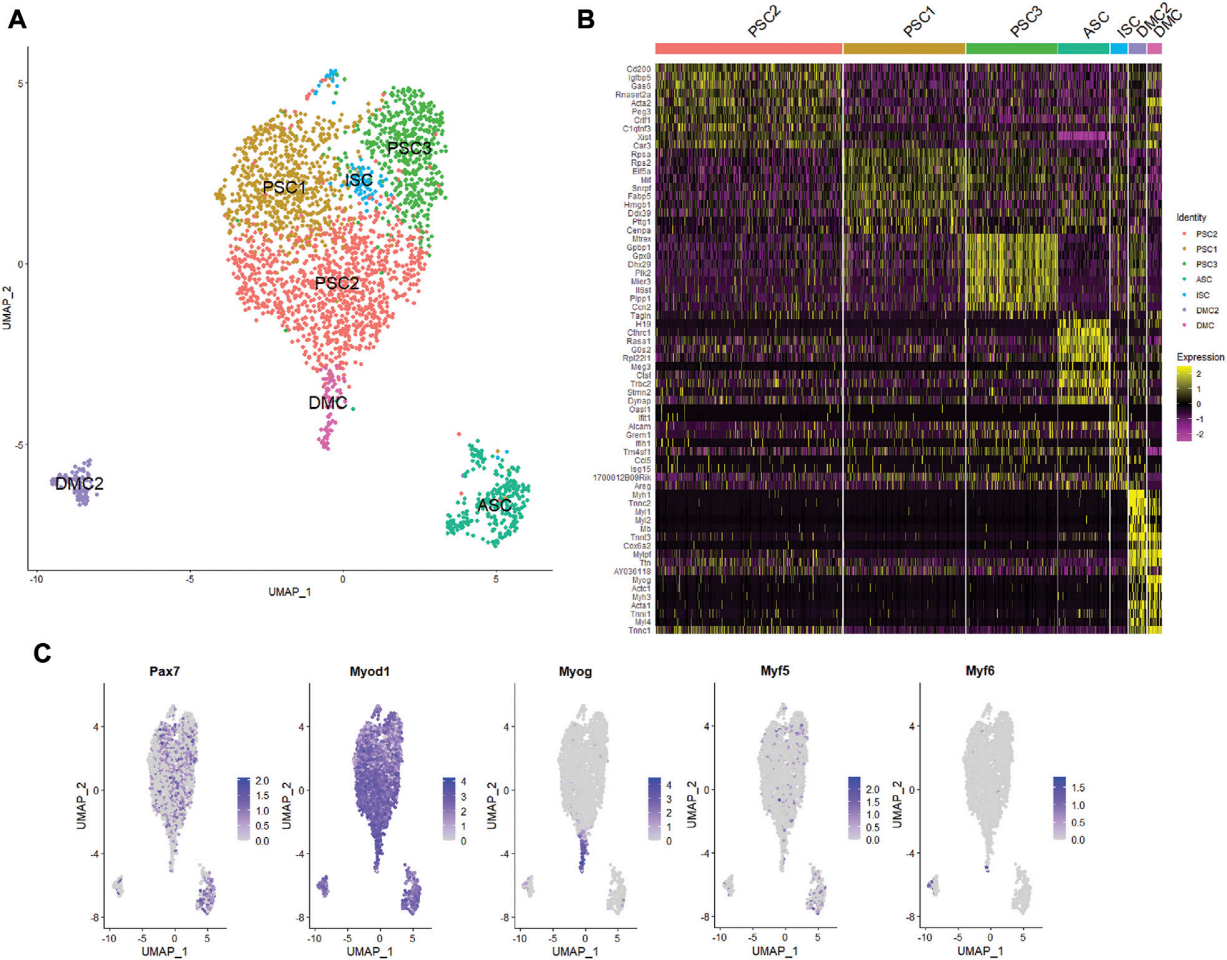
Single cell RNA-seq analysis of primary mouse MuSC culture. **(A)** UMAP clustering of the PBS-treated MuSCs. Cells clustered into seven groups based on differential gene transcription. **(B)** Heatmap depicting top 10 differentially expressed genes for each cluster. **(C)** Feature plots show the distribution of the myogenic transcription factors of *Pax7, Myod1, Myog, Myf5,* and *Myf6* in the MuSCs.

**FIGURE 3:**
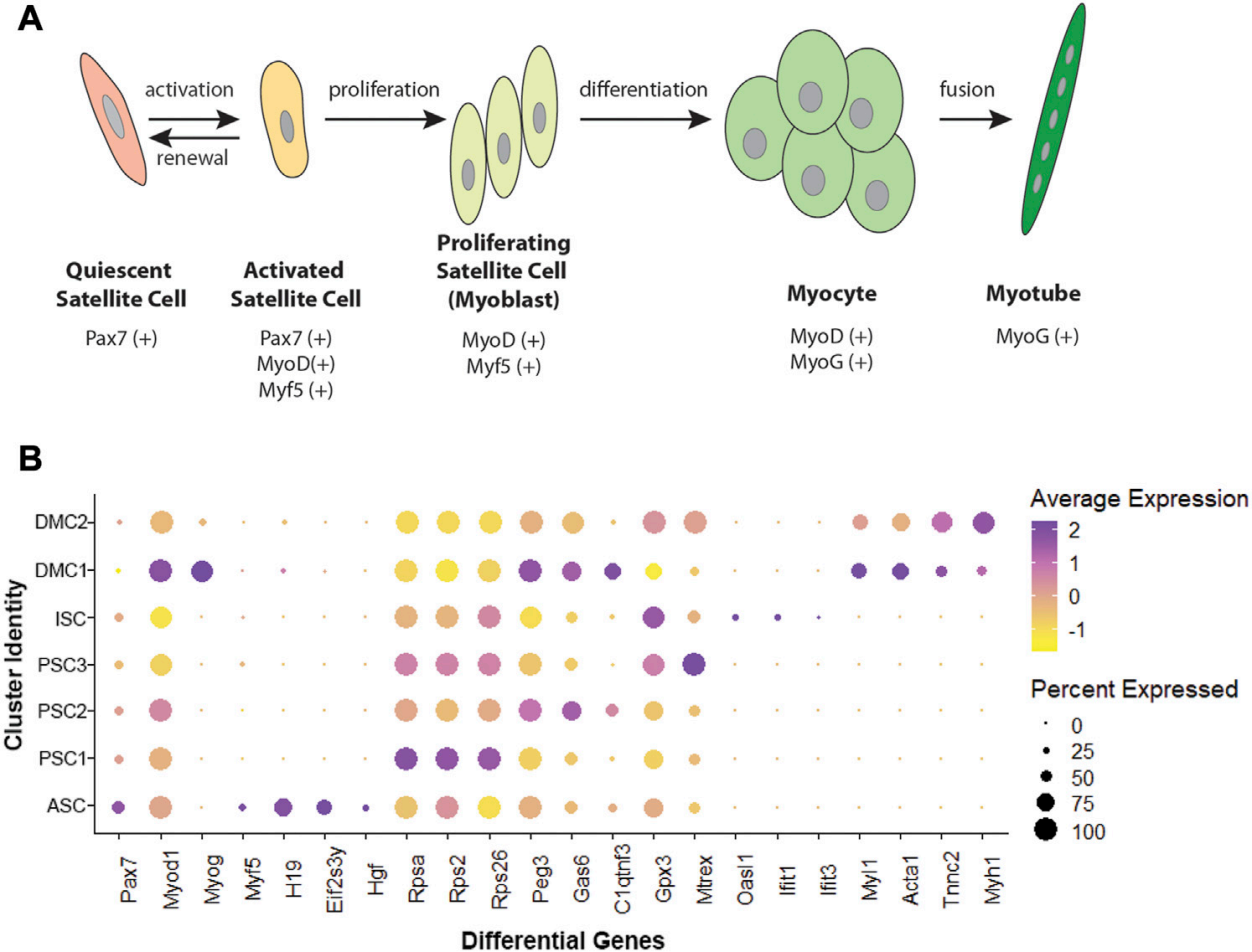
Myogenic transcription factors and differentially expressed genes in MuSCs. **(A)** Schematic of satellite cell progression to differentiated myotube, including the myogenic transcription factor profile for each stage. **(B)** Dotplot showing average and percent expression of genes that differentiate the 7 cell clusters in PBS-treated MuSCs.

Unsupervised clustering and UMAP embedding was performed using the Seurat R package and identified seven unique clusters in PBS-treated MuSCs based on differentially expressed genes (Figures 2A,B; Supplementary Table S2). All clusters were determined to consist of myogenic lineage cells based on the expression of one or more of the muscle-specific transcription factors, *Pax7*, *Myod1*, *Myog*, and *Myf5* (Figure 2C), known to have dynamic expression in satellite cells as they progress along the continuum from quiescent satellite cell through activation, proliferation, and differentiation (Figure 3A). The pattern of transcription of these four factors and other differentially expressed genes was used to identify the cell clusters within the PBS-treated MuSC culture (Figure 3B). Activated satellite cells (ASC) were identified based on transcription of *Pax7* and the satellite cell activation and proliferation markers *H19, EIF2S3Y*, and *HGF* (Sheehan et al., 2000; Li et al., 2016; Martinet et al., 2016). The vast majority of the cells sorted into 3 cell clusters, PSC1—PSC3, with a similar pattern of reduced *Pax7,* and increased *Myod1,* transcription. PSC1 had a marked increase in ribosomal protein expression (*Rspa, Rps2, Rps26,* and *Rps29*)*,* necessary for ribosome biogenesis and post-transcriptional regulation. Expression of these genes is associated with the increase in protein production needed for the transition of ASCs to proliferating myoblasts (Chaillou et al., 2017; Gayraud-Morel et al., 2018). The second and largest cell cluster, PSC2, was enriched for markers of proliferating myoblasts, including *Peg3*, *Gas6*, and *C1qtnf3* (Correra et al., 2018; Mervis et al., 2020; Otani et al., 2020). The third PSC cluster (PSC3) had a higher expression level of glutathione peroxidase 3 (*Gpx3*) and the RNA helicase *Mtrex,* implying that these cells were responding to local environmental stressors that may not be related to the normal progression of satellite cells (El Haddad et al., 2012). There was a small cell population related to the PSC cell clusters, except for reduced transcription of *Myod1* and *Myog* and enrichment for genes in the antiviral IFN pathway and downstream interferon stimulated genes (ISG), including *Oasl1, Ifit1*, *Ifit3*, and *Rsad2* (Figure 3B). Thus, we have designated this cluster, IFN stimulated cells or ISCs. Finally, two clusters of differentiating myocytes (DMC1 and 2) were present in the culture, based on the expression of *Myog* and *Myod1,* as well as sarcomeric proteins including *Myl1*, *Acta1*, *Tnnc2*, and *Myh1*. Thus, the PBS treated primary satellite cell culture was comprised of cells along the continuum from activated satellite cells to differentiating myocytes with several subsets of myoblasts with distinct patterns of gene transcription.

### 3.3 LPS induces chemokines in primary MuSCs

**FIGURE 4:**
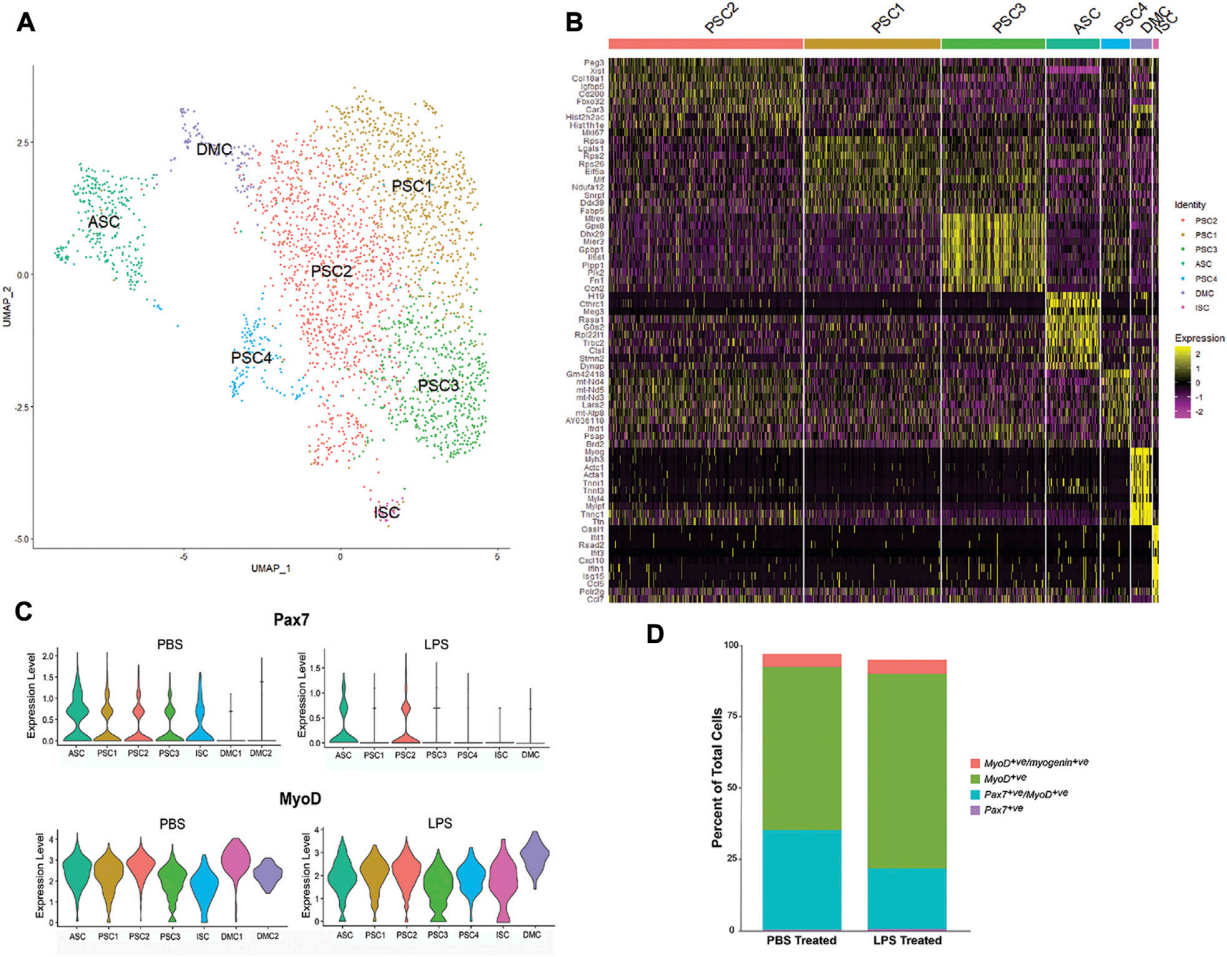
Single-cell RNA-SEQ analysis of LPS-treated primary mouse MuSCs. **(**A) UMAP clustering of the LPS-treated MuSC. Cells cluster into seven groups based on differential gene transcription. **(B)** Heatmap depicting top 10 differentially expressed genes for each cluster. **(C)** Violin plots of *Pax7* and *Myod1* transcription in PBS and LPS treated MuSCs. **(D)** The distribution of satellite cells (*Pax7* ^*+ve*^ only), activated satellite cells (*Pax7* ^*+ve*^ */Myod1* ^*+ve*^), proliferating myoblasts (*Myod1* ^*+ve*^ only) and differentiating myocytes (*Myod1* ^*+ve*^ */Myog* ^*+ve*^) in PBS and LPS treated cultures. Cell populations are presented as percent of the total population.

To extend our evaluation of LPS induction of cytokines and chemokines in muscle (Figure 1), MuSC cultures were treated with LPS (1 mg/mL) for 2 h and profiled by scRNA-Seq. Unsupervised clustering and UMAP embedding was performed using the Seurat R package under the same conditions as the control culture (Figure 4, Supplementary Table S3). A similar set of cell clusters, as described above, were identified based on differential gene expression. However, there were exceptions, including a fourth PSC cluster (PSC4) and the absence of the second DMC cluster. The PSC4 cluster was distinguished by decreased expression of more than 20 ribosomal proteins. The significance of this reduction of the translational machinery is not clear, though there is evidence of LPS reducing protein synthesis in muscle tissue (Gordon et al., 2013).

The level of transcription of the myogenic markers, *Pax7* and *Myod1,* was examined in the cell clusters of PBS and LPS treated cells (Figure 4C). *Pax7* transcription was reduced in PSC1, PSC3 and ISC clusters and reduced in the ASC cluster. In contrast, *Myod1* transcription levels are unaffected by LPS treatment. Alternatively, the distribution of cells along the satellite cell continuum was evaluated independent of cell clusters by comparing the number of cells co-expressing the myogenic markers, *Pax7*, *Myod1*, and *Myog* (Figure 4D). While the number of *Pax7* ^*+ve*^-only satellite cells and *Myod1* ^*+ve*^ */Myog* ^*+ve*^ differentiating myocytes remained relatively constant, there was an expansion of the *Myod1* ^*+ve*^ myoblasts cells at the cost of the *Pax7* ^*+ve*^ */Myod1* ^*+ve*^ activated satellite cells. This is consistent with the reduction of *Pax7* transcription after LPS treatment. It remains to be determined if this is due to direct regulation by LPS signaling, or the indirect result of promoting proliferation of myoblasts through cytokine production.

**FIGURE 5:**
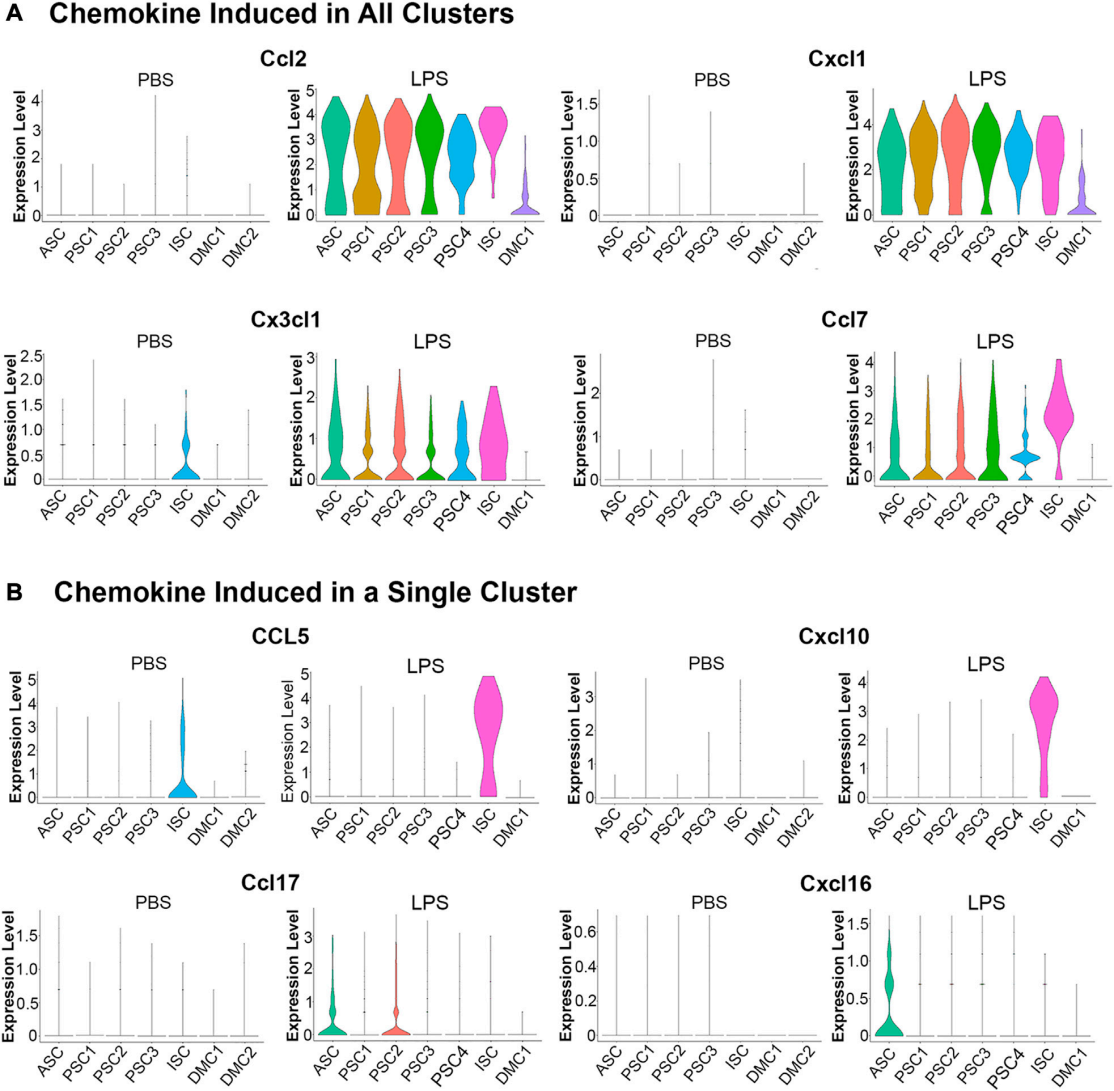
Violin plots of chemokine transcription in PBS and LPS treated MuSCs. **(A)** The transcription of chemokines *Ccl2, Cxcl1, Cx3cl1* and *Ccl7* were upregulated in all cluster after LPS treatment. **(B)** The transcription of chemokines *Ccl5, Cxcl10, Ccl17* and *Cxcl16* were upregulated in a singular cluster post-LPS treatment. Expression is depicted as Log 2 fold change above average expression across all cells.

An evaluation of chemokine production in response to LPS by scRNA-Seq showed that 13 members of the C-C and C-X-C subfamilies (*Ccl2, Ccl5, Ccl7, Ccl17, Ccl20, Ccl27a, Cxcl1, Cxcl2, Cxcl4, Cxcl10, Cxcl12, Cxcl16,* and *Cx3cl1*) were expressed in MuSCs. A comparison of transcription of these factors across the cell clusters in the LPS and PBS control conditions revealed two distinct patterns (Figure 5). The first pattern included chemokines not expressed in the control cells and broadly transcribed after treatment with LPS (*e.*g., *Ccl2, Cxcl1, Ccl7,* and *Cx3c1*). This is in contrast to chemokines that were induced in a single cell cluster. This was observed for *Ccl5* and *Cxcl10*, which were induced in the ISC cluster, and *Ccl17* and *Cxcl16* in the ASC cluster (Figure 5B). *Ccl5* and *Cxcl10* are induced by type I IFNs (Kelly-Scumpia et al., 2010; Nakano et al., 2012) consistent with our designation of this cluster as IFN stimulated.

LPS induction of chemokines was validated in independently isolated primary MuSCs by RT-qPCR of total RNA using gene specific primers. Transcripts for the chemokines *Ccl2, Ccl5, Ccl7, Cxcl1*, and *Cxcl10* were significantly higher in LPS-treated cells as compared to the PBS-treated controls (Supplementary Table S4). Overall, these studies revealed that MuSCs can be induced to express many C-C and C-X-C chemokines with LPS and they are differentially expressed across the cell clusters. ASC and ISC cell clusters express separate sets of chemokines, raising the possibility that they serve unique functions during muscle inflammation response.

**FIGURE 6:**
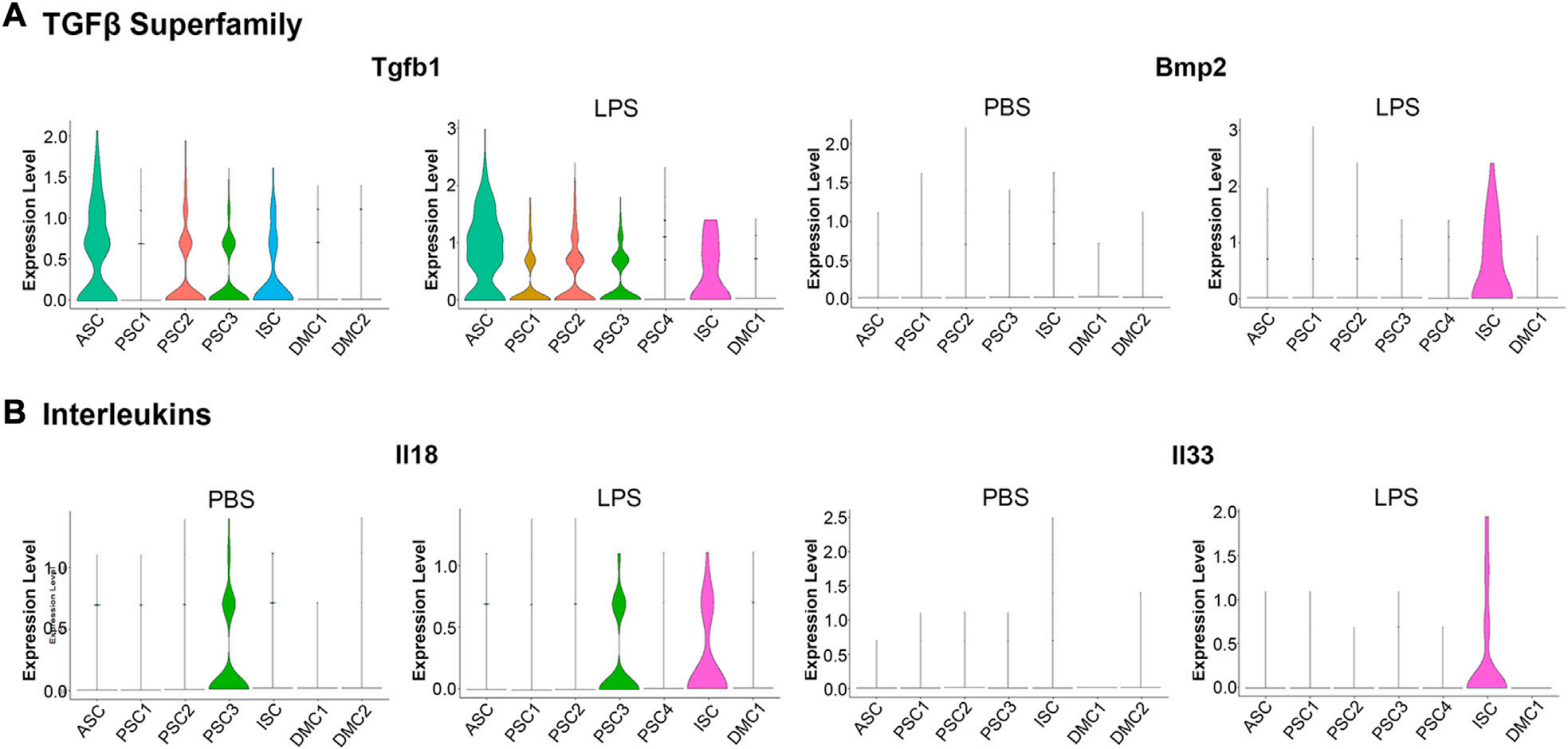
Violin plots of cytokine transcription in PBS and LPS treated MuSCs. **(A)** The transcription pattern of the TGFβ superfamily genes *Tgfb1* and *Bmp2*. **(B)** The transcription pattern of the Interleukins superfamily genes *Il18* and *Il33*. Expression is depicted as Log 2 FC above average expression across all cells.

An examination of proinflammatory cytokine transcription in response to LPS treatment revealed a modest induction of a limited number of factors when compared to the robust induction of chemokines (Figure 5). Among the TGFβ superfamily, known to positively regulate cells of the innate immune system (Chen and ten Dijke, 2016), *Tgfb1* was constitutively transcribed at low levels in the ASC, PSC2, PSC3, and ISC clusters. With LPS treatment, *Tgfb1* was induced in PSC1 cells (Figure 6A). Similarly, *Tgfb2* was induced in PSC1 and PSC2 cells, but constitutively expressed in DMCs (data not shown). In contrast, *Bmp2*, a known antiviral ISG in bone marrow macrophages (Liu et al., 2012), was induced selectively in the ISC cluster (Figure 6A). Interestingly, *Il18* and *Il33*, members of the IL-1 family of proinflammatory cytokines (Chan et al., 2019; Martinon et al., 2009), were weakly induced in ISCs (Figure 6B). Overall, the MuSCs expressed cytokines and chemokines in a heterogeneous pattern in response to inflammatory signals.

### 3.4 LPS induces the interferon signaling pathway in the ISC cell cluster

Single cell sequencing of the PBS control cells revealed a low level expression of components of the IFN signaling pathway in the ISC cluster (Figure 2). LPS induced the expression of *Ccl5, Cxcl10, Il18,* and *Il33* in these cells (Figures 5, 6), supporting a proinflammatory role for this cluster. Since LPS acts through the TLR4 receptor to induce IFN *α* and *γ* expression (Richez et al., 2010; Wang et al., 2017), transcription of members of the antiviral IFN pathway were examined (Table 1). Of note, two cytosolic sensors of viral DNA and RNA, *Ifih1*/*MDA-5* and *Ddx58*/*Rig-1* (Loo and Gale, 2011), as well as their downstream transcription factor, *Irf7*, were selectively induced in the ISC cells. Further, twelve other IFN induced signaling factors associated with antiviral activity were also induced (Table 1). It should be noted that IFN-α, -β, or -γ transcription was not detected, raising the possibility that the antiviral IFN pathway was activated through the cytosolic receptors independent of the IFNs.

**TABLE 1:** Induction of genes in the antiviral IFN pathway in the ISC cluster of PBS and LPS treated MuSCs. Transcription of genes reported to participate in the antiviral IFN pathway or be downstream induced genes were quantified by scRNA-Seq and expressed as Log2 fold change above average expression across all cells in the PBS- or LPS-treated MuSC library.

IFN stimulated genes	PBS treated MuSC	LPS treated MuSC
	avg log2 FC	avg Log2 FC
*Intracellular Sensors*		
Ifih1	1.3457	2.6062
Ddx60	0.2366	0.8932
Ddx58	0.2047	0.4750
*IFN Regulatory Transcription Factors*		
Irf7	0.1605	0.3152
*IFN Inducible Chemokines*		
Ccl5	2.3052	4.3164
Cxcl10	1.5864	4.1414
*IFN Inducible Host Defense Genes*		
Ifit1	3.0999	4.7017
Isg15	2.5953	4.4797
Rsad2	1.2518	3.4335
Oasl1	1.5689	2.9600
Oasl2	0.5788	1.5041
Bmp2	0.2401	1.0713
Apol9a	0.3192	0.9115
Ifit3b	0.2866	0.8729
Apol9b	0.2094	0.8477
Ifit2	0.5599	0.8431
Gbp3	0.1478	0.6641
Rtp4	0.1878	0.4956

**FIGURE 7:**
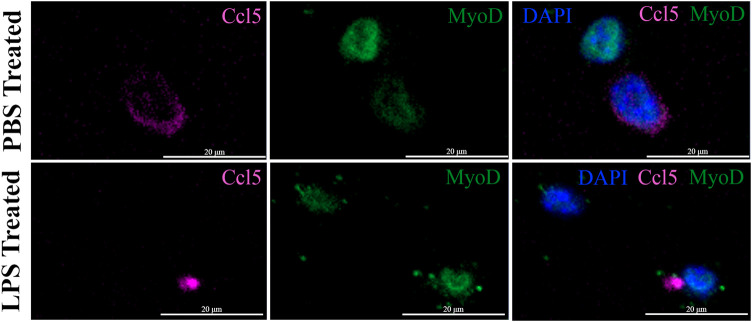
Co-expression of CCL5 and MYOD in proliferating MuSCs treated with LPS and PBS. The expression of CCL5 (magenta) and MYOD (green) was detected by indirect IF in MuSCs treated with LPS (1 mg/mL) or control PBS, for 2 h or control PBS treatment. CCL5 can be detected in the cytoplasm and MYOD in the nucleus. The nuclei were visualized with DAPI (blue). Images were taken at ×20 magnification. Scalebar = 20 µm.

The expression of the IFN pathway marker, CCL5, in a small subset of MuSCs was confirmed by IF done using antibodies specific to both MYOD and CCL5. CCL5^+ve^/MYOD^+ve^ cells were detected in newly isolated MuSCs from the *quadriceps femoris* of 3 month old mice treated with LPS or PBS (Figure 7). Double positive cells represented 2.3% of the LPS treated MuSCs, which was comparable to the distribution (2.08%) of *Myod1* positive cells expressing ISG genes identified in the scRNA-Seq analysis (Table 2). These genes were also detectable in PBS treated control cultures (Figure 7), indicating that activation of the ISG gene expression was independent of LPS activation, but the expression level was increased in response to this inflammatory signal.

**TABLE 2:** Distribution of MuSCs with the activated antiviral IFN pathway in PBS- and LPS-treated cells. Representation of the ISC cluster within the control and LPS treated MuSCs was evaluated by co-expression of *Isg15*, *Ifih1*, Oasl, *Ifit1*, and *Myod1* as measured by scRNA-Seq. ISC cells were independently assess in culture by co-expression of CCL5 and MYOD by IF.

	Single cell RNA-Seq			Immunostaining		
	Transcription of antiviral IFN pathway			CCL5/MYOD positive cells		
Treatment	+^ve^ Cells	Total Cells	%	+^ve^ Cells	Total Cells	%
PBS	72	2,751	2.61	49	3,600	1.36
LPS	58	2,801	2.08	97	4,200	2.3

### 3.5 Induction of IFN pathway in injured muscle

**FIGURE 8:**
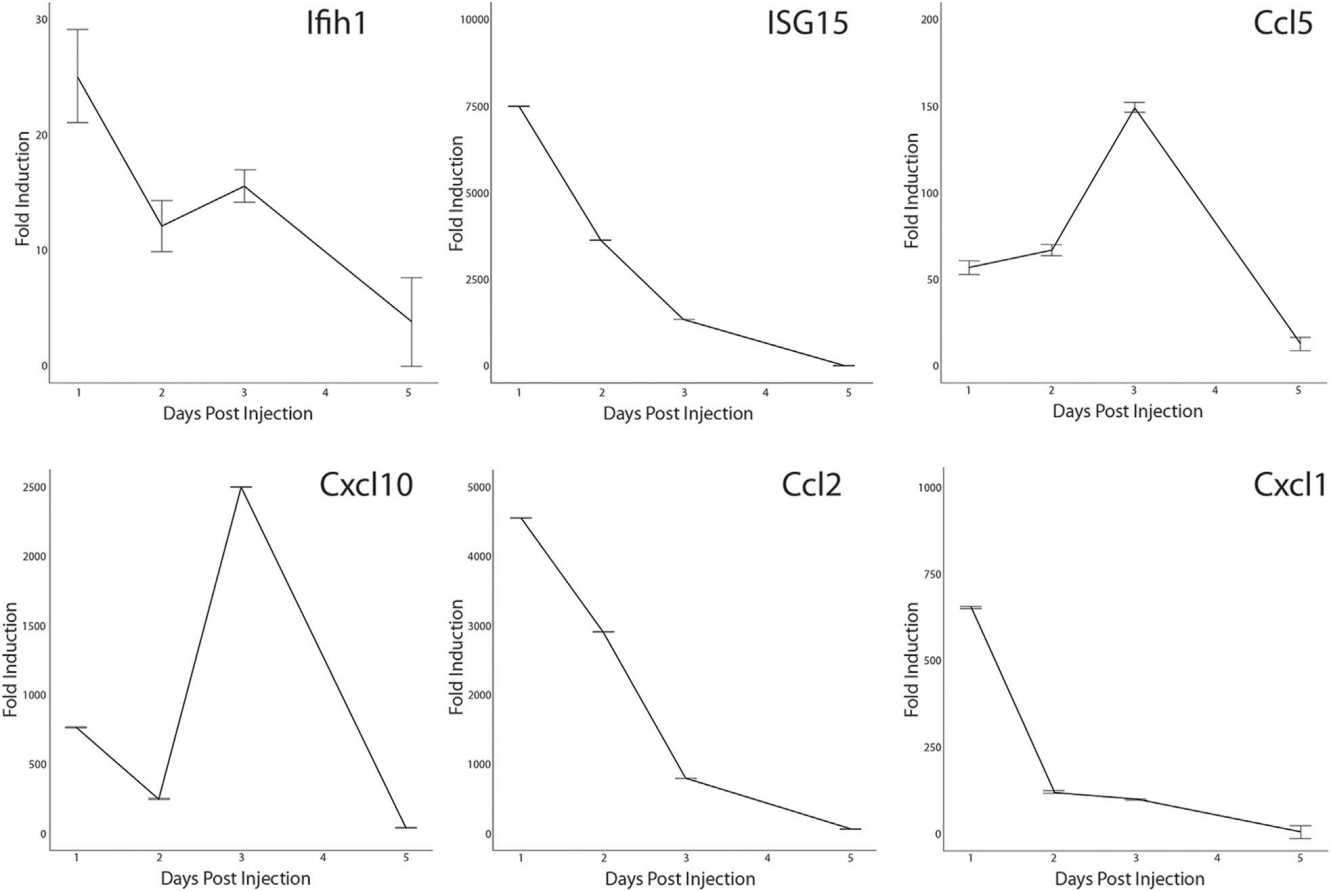
Transcription of antiviral interferon pathway genes in CTX-injured *quadriceps.* CTX-injured *quadriceps* had total RNA isolated at 1, 2, 3, and 5 DPI. Transcripts of *Ifih1, Isg15*, *Ccl5, Cxcl10, Ccl2*, and *Cxcl1* were quantified by RT-qPCR. RNA transcript levels are expressed as the fold induction over RNA isolated from the uninjured contralateral leg. Significance was determined by Student’s t-test, all timepoints *p* < 0.0001 except for *Cxcl1*, *Ifih1*, and *Ccl5* at 5 DPI.

Activation of the antiviral IFN pathway is associated with the chronic muscle inflammation noted in DM patients and IFN-γ has been linked to muscle regeneration (Greenberg et al., 2005; Cheng et al., 2008; Suárez-Calvet et al., 2014). Since we observed that the IFN pathway was activated in a small percentage of MuSCs isolated from healthy mice, it was possible that it has a role in skeletal muscle repair as well. We further examined if we could detect activation of a subset of the ISG in response to acute injury. CTX was injected into the *quadriceps femoris* of Bl10 mice and total RNA isolated from the site of injury at 1, 2, 3, and 5 days post-injury (DPI) and from the uninjured contralateral leg. RT-qPCR was done using gene specific primers. When compared to the uninjured muscle, both *Ifih1* and *Isg15* transcription were rapidly induced at 1 DPI followed by a gradual decline. In contrast, expression of the IFN-regulated chemokines, *Ccl5* and *Cxcl10*, peaked at 3 DPI before sharply declining (Figure 8). This is distinctly different from the broadly transcribed chemokines, *Ccl2* and *Cxcl1*, that peaked earlier at 1 DPI. Thus, these data demonstrate that components of the antiviral IFN pathway originally observed with LPS treatment are expressed *in vivo* in response to injury also.

**FIGURE 9:**
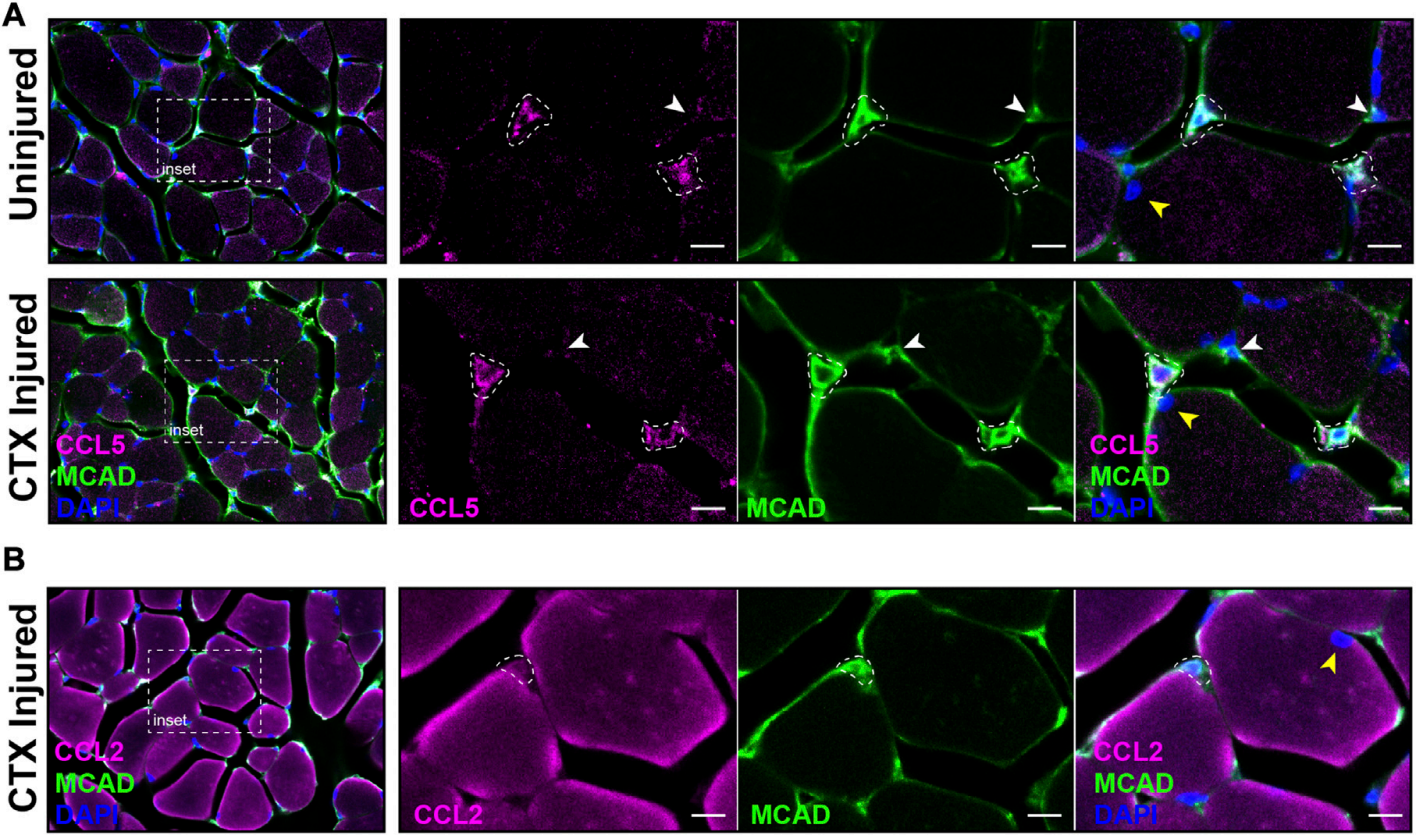
Co-expression of CCL2, CCL5 and MCAD in CTX-injured skeletal muscle. **(A)** The expression of CCL5 (magenta) and MCAD (green) were visualized in DAPI-labeled (blue) CTX-injured and uninjured *quadriceps* at 3 DPI by IF. White dotted outlines mark CCL5/MCAD co-expressing satellite cells, the white arrowhead indicates a MCAD-only expressing satellite cell, and the yellow arrowhead marks a muscle fiber nucleus. **(B)** CCL2 (magenta) and MCAD (green) co-expressing satellite cells (white dotted outline) were detected in CTX-injured *quadriceps* at 1 DPI. The nuclei were labeled with DAPI (blue). In contrast to CCL5, significant expression of CCL2 was detected in muscle fibers. The yellow arrowhead denotes a muscle fiber nucleus. Scalebar = 10 µm.

The distribution of satellite cells that had the antiviral IFN pathway activated in muscle was evaluated by IF with antibodies specific for CCL5 and M-Cadherin (MCAD), a cell adhesion protein expressed on satellite cells in their niche and proliferating myoblasts. MCAD expression is used to distinguish the mononuclear satellite cells from the adjacent muscle fiber (Yin et al., 2013). IF was performed on sections of CTX injured and contralateral control *quadriceps*. Analysis demonstrated that CCL5^+ve^/MCAD^+ve^ cells represented 10.18% of total MCAD^+ve^ satellite cells of uninjured muscle and 10.52% of the total MCAD^+ve^ cells in CTX injured muscle at 3 DPI (Figure 9A). In comparison, we found CCL2, which was transcribed at high levels in LPS-induced MuSCs, was broadly expressed in both satellite cells and intact muscle fibers at 1 DPI (Figure 9B).

## 4 Discussion

In this study, we re-evaluated the muscle-immune paradigm during muscle damage and repair. The transcription of immune effector genes has been reported in muscle through a MYF6-dependent pathway (Barruet et al., 2020; Lazure et al., 2020). However, their capacity to be induced to express pro-inflammatory genes in response to infection or injury remains less clear. Here we leveraged primary satellite cell cultures and single cell RNA sequencing technology to examine the capacity of satellite cells and myoblasts to express cytokines and chemokines. Here, we demonstrated that non-sterile PAMPs (LPS) induced a heterogeneous pattern of expression of proinflammatory cytokines and chemokines (Figures 3–6). Single-cell RNA-SEQ revealed that proliferating MuSCs in culture were comprised of distinct clusters that ranged from activated satellite cells, proliferating myoblasts, to differentiating myocytes (Figures 2, 3). Though many of the chemokines and cytokines were broadly expressed across these clusters, there were examples of selective transcription in ISC, ASC, and PSC1. Thus, the responsiveness of the MuSCs to inflammatory signals can vary dependent on a cell’s myogenic status on the pathway towards differentiation. Of particular interest are the ISCs that appear to have primed the antiviral IFN pathway which then can be activated by LPS to selectively increase the expression of *Ccl5, Cxcl10*, *Il18,* and *Il33*. There are descriptions of satellite cell subsets predicted to have an adaptive response for injury and stress (G_Alert_) (Rodgers et al., 2014; Scaramozza et al., 2019; De Micheli et al., 2020). Our data is the first indication that a subgroup of satellite cells that could be acting as sensors for infection and injury in the muscle.

An evaluation of the chemokines and cytokines expressed in response to LPS predicts that MuSCs can promote the proinflammatory environment in muscle. Many of these factors have been reported to recruit neutrophils, monocytes, and T cells to the site of muscle injury, including CCL2, CCL5, CCL7, CXCL16, and CX3CL3 (Figure 5; Zhang et al., 2009; Martinez et al., 2010; Crescioli et al., 2012; Kohno et al., 2011; Strömberg et al., 2016). Further, our finding that CCL2 and CCL5 were expressed in satellite cells and muscle fibers at the site of CTX-induced injury, indicated that induction occurs *in vivo* and this response is not restricted to LPS. Several of the chemokines expressed in LPS-treated MuSC are predicted to participate in the expansion of the satellite cell population during skeletal muscle repair. CCL2 and CXCL16 promote satellite cell proliferation (Yahiaoui et al., 2008; Zhang et al., 2009), while CXCL1, CXCL10, and CCL17, negatively regulate myoblast differentiation, preventing premature exit from the cell cycle (Ge et al., 2013; Hogan et al., 2018). Thus, the pattern of expression is consistent with a dual role for MuSCs during regeneration, recruiting the innate immune cells and promoting myoblast expansion.

An unexpected outcome of the scRNA-Seq analysis was the identification of a small population of MuSCs with the antiviral IFN pathway activated in the absence of LPS. Approximately, 2% of the proliferating MuSCs in culture and 10% of satellite cells in muscle tissue expressed genes associated with the IFN-independent early antiviral components of the pathway that sense cytosolic viral DNA and double stranded RNA. Among the IFN genes expressed were the cytosolic sensors, *Ddx58* and *Ifih1*, which bind the triphosphate group at the 5’ end of viral RNA (Wu and Chen, 2014). This interaction induces expression of type I IFNs and a subset ISG proteins mediated by the transcription factors nuclear factor κ-light-chain enhancer of activated B cells (NF-κB), and the IFN regulatory factors IRF3 and IRF7 (Grandvaux et al., 2002). Among the antiviral pathway genes detected at high levels in uninduced MuSC ISCs, *Oasl, OasII, Isg15*, *Rsad2,* and *Irf7,* are positive regulators of IFIH1/DDX58-dependent signaling (Kim et al., 2008; MacMicking et al., 2012; Hee and Cresswell, 2017; Li et al., 2017; Liu et al., 2021). Transcription of these genes in the PBS treated controls suggests these cells are maintained in a primed state. How the antiviral pathway is activated at low levels in the absence of a viral infection is unclear. Further, the presence of satellite cells with activated antiviral IFN pathway 3 days after a sterile muscle injury reinforces the presence of a mechanism of activation that is virus independent. One possible explanation comes from the observation that the endoribonuclease, RNase L, and the 2′,5′-oligoadenylate synthetase, OAS, can produce small self-RNAs from cellular mRNA that is recognizable by IFIH1 and DDX58 (Malathi et al., 2007; Stok et al., 2020). This suggests that ISCs are capable of self-priming or possibly detecting RNA from adjacent damaged cells post-injury in the absence of infection.

Expression of the IFN signaling pathway in satellite cells has been previously described in patients with dermatomyositis (DM) and polymyositis, demonstrating the capacity of muscle to regulate this pathway (Greenberg et al., 2005; Salajegheh et al., 2010; Suárez-Calvet et al., 2014). This suggests a mechanism for the autoimmune pathology of DM patients through overstimulation of IFIH1/DDX58 sensors by self-RNA. Consistent with this, muscle from these DM patients express type I IFN and upregulate *Isg15* and other ISG genes at levels approaching 100 fold higher than unaffected muscle (Baechler et al., 2011). Interestingly, activation of the IFN pathway also was detected in uninjured human *vastus lateralis* satellite cells by scRNA-Seq (Barruet et al., 2020). They identified a small population of *Pax7* ^+ve^ cells that expressed many of the IFN genes described in our study. It should be noted that the chemokines, CCL5 and CXCL10, were not observed in the human satellite cells, whereas our data demonstrated expression of *Ccl5* and *Cxcl10* transcripts in cultured satellite cells, and CCL5 protein in muscle. As our current studies demonstrate that IFN pathway and chemokine expressing MuSCs are present and expression of these genes can be upregulated by LPS, we have an opportunity for further study of the role of *Ccl5* and IFN pathway genes in muscle inflammation.

Heterogeneity of the adult satellite cell population has been well studied as it relates to tissue origin, cell cycle, and function in skeletal muscle repair (Biressi and Rando, 2010). In this study, we expand our view of heterogeneity to include the capacity of MuSCs to produce chemokines and cytokines that act on the myeloid and myogenic cells present at the site of injury. *Pax7* ^+ve^ satellite cells (ASC) selectively expressed *Ccl17* and *Cxcl16* that promoted cell proliferation in myogenic cells, as well recruiting neutrophils and macrophages. Perhaps more surprising, was the identification of ISC population, present in both treated and control MuSC cultures and in the satellite cells of injured muscle. Their presence in the absence of injury or inflammatory signals suggests that they serve as sentinel cells. The cells are specifically predisposed to activate the IFIH1/DDX58-dependent pathway. This is underscored by the results of our LPS treatment of MuSCs. LPS induces a broad set of proinflammatory cytokines and chemokines in immune and non-immune cells through a TLR4-dependent manner, including the IFIH1/DDX58-dependent pathway (Lu et al., 2008; Imaizumi et al., 2013). While LPS induced transcription of *Ccl2, Ccl7,* and *Cxcl1* in all cell clusters, induction of *Ifih1, Ddx58, Ccl5* and *Cxcl10* transcription was restricted to the ISC cluster. The nature of the signals regulating the heterogeneity of MuSC response to LPS or other immunogens remains to determined.

Overall, our studies demonstrate that satellite cells and proliferating myoblasts are competent to respond to PAMPs and DAMPs by expressing of chemokines and cytokines. Functionally, this is likely a combination of amplifying the signals at the site of injury that recruit and activate proinflammatory immune cells and autocrine signaling promoting the rapid expansion of the satellite cells prior to differentiation into muscle fibers. These studies suggest that the antiviral IFN pathway plays a greater role in sterile inflammation in response to muscle damage than previously considered. Additional studies are needed to understand its contribution to the regulation of the innate immune response and satellite cell proliferation and differentiation.

## Data Availability

The datasets presented in this study can be found in online repositories. The names of the repository/repositories and accession number(s) can be found below: https://www.ncbi.nlm.nih.gov/geo/, GSE216848.

## References

[B1] Baechler E. C. Bilgic H. Reed A. M. (2011). Type I interferon pathway in adult and juvenile dermatomyositis. Arthritis Res. Ther. 13 249, 249. 10.1186/ar3531 22192711 PMC3334651

[B2] Barruet E. Garcia S. M. Striedinger K. Wu J. Lee S. Byrnes L. (2020). Functionally heterogeneous human satellite cells identified by single cell RNA sequencing. eLife 9, e51576. 10.7554/eLife.51576 32234209 PMC7164960

[B3] Biressi S. Rando T. A. (2010). Heterogeneity in the muscle satellite cell population. Semin. Cell Dev. Biol. 21, 845–854. 10.1016/j.semcdb.2010.09.003 20849971 PMC2967620

[B4] Bivona J. J. III Crymble H. M. Guigni B. A. Stapleton R. D. Files D. C. Toth M. J. (2021). Macrophages augment the skeletal muscle proinflammatory response through TNFα following LPS-induced acute lung injury. FASEB J. 35, e21462. 10.1096/fj.202002275RR 33724561 PMC7970444

[B5] Boehm U. Klamp T. Groot M. Howard J. C. (1997). Cellular responses to interferon-gamma.Annu. Rev. Immunol. 15, 749–795. 10.1146/annurev.immunol.15.1.749 9143706

[B6] Brigitte M. Schilte C. Plonquet A. Baba-Amer Y. Henri A. Charlier C. (2010). Muscle resident macrophages control the immune cell reaction in a mouse model of notexin-induced myoinjury. Arthritis Rheum. 62, 268–279. 10.1002/art.27183 20039420

[B7] Chaillou T. Kirby T. J. McCarthy J. J. (2017). Ribosome biogenesis: Emerging evidence for a central role in the regulation of skeletal muscle mass. J. Cell. Physiol. 229, 1584–1594. 10.1002/jcp.24604 PMC4868551 24604615

[B8] Chan B. C. L. Lam C. W. K. Tam L.-S. Wong C. K. (2019). IL33: Roles in allergic inflammation and therapeutic perspectives. Front. Immunol. 10, 364. 10.3389/fimmu.2019.00364 30886621 PMC6409346

[B9] Chargé S. B. Rudnicki M. A. (2004). Cellular and molecular regulation of muscle regeneration. Physiol. Rev. 84, 209–238. 10.1152/physrev.00019.2003 14715915

[B10] Chen S. E. Gerken E. Zhang Y. Zhan M. Mohan R. K. Li A. S. (2005). Role of TNF-{alpha} signaling in regeneration of cardiotoxin-injured muscle. Am. J. Physiol. Cell Physiol. 289, C1179–C1187. 10.1152/ajpcell.00062.2005 16079187 PMC3099530

[B11] Chen W. ten Dijke P. (2016). Immunoregulation by members of the TGFβ superfamily. Nat. Rev. Immuno 16, 723–740. 10.1038/nri.2016.112 27885276

[B12] Cheng M. Nguyen M.-H. Koh T. J. (2008). Endogenous interferon-gamma is required for efficient skeletal muscle regeneration. Am. J. Physiol. Cell Physiol. 294, C1183–C1191. 10.1152/ajpcell.00568.2007 18353892

[B13] Ciesielska A. Matyjek M. Kwiatkowska K. (2021). TLR4 and CD14 trafficking and its influence on LPS-induced pro-inflammatory signaling. Cell Mol. Life Sci. 78, 1233–1261. 10.1007/s00018-020-03656-y 33057840 PMC7904555

[B14] Collins R. A. Grounds M. D. (2001). The role of tumor necrosis factor-α (TNFα) in skeletal muscle regeneration. Studies in TNFα ^−/−^ and TNFα ^−/−^/LTα ^−/−^ mice. J. Histochem Cytochem 49, 989–1001. 10.1177/002215540104900807 11457927

[B15] Correra R. M. Ollitrault D. Valente M. Mazzola A. Adalsteinsson B. T. Ferguson-Smith A. C. (2018). The imprinted gene Pw1/Peg3 regulates skeletal muscle growth, satellite cell metabolic state, and self-renewal. Sci. Rep. 8, 14649. 10.1038/s41598-018-32941-x 30279563 PMC6168517

[B16] Crescioli C. Sottili M. Bonini P. Cosmi L. Chiarugi P. Romagnani P. (2012). Inflammatory response in human skeletal muscle cells: CXCL10 as a potential therapeutic target. Eur. J. Cell Biol. 91, 139–149. 10.1016/j.ejcb.2011.09.011 22176919

[B17] De Michelli A. J. Spector J. A. Elemento O. Cosgrove B. D. (2020). A reference single-cell transcriptomic atlas of human skeletal muscle tissue reveals bifurcated muscle stem cell populations. Skelet. Muscle 10, 19. 10.1186/s13395-020-00236-3 32624006 PMC7336639

[B18] De Rossi M. Bernasconi P. Baggi F. de Waal Malefyt R. Mantegazza R. (2000). Cytokines and chemokines are both expressed by human myoblasts: Possible relevance for the immune pathogenesis of muscle inflammation. Int. Immunol. 12, 1329–1335. 10.1093/intimm/12.9.1329 10967028

[B19] Dogra C. Changotra H. Mohan S. Kumar A. (2006). Tumor necrosis factor-like weak inducer of apoptosis inhibits skeletal myogenesis through sustained activation of nuclear factor-kappaB and degradation of MyoD protein. J. Biol. Chem. 281, 10327–10336. 10.1074/jbc.m511131200 16461349

[B20] Dogra C. Changotra H. Wedhas N. Qin X. Wergedal J. E. Kumar A. (2007). TNF-related weak inducer of apoptosis (TWEAK) is a potent skeletal muscle-wasting cytokine. FASEB J. 21, 1857–1869. 10.1096/fj.06-7537com 17314137 PMC4154373

[B21] El Haddad M. Jean E. Turki A. Hugon G. Vernus B. Bonnieu A. (2012). Glutathione peroxidase 3, a new retinoid target gene, is crucial for human skeletal muscle precursor cell survival. J. Cell Sci. 125, 6147–6156. 10.1242/jcs.115220 23132926

[B22] Fielding R. A. Manfredi T. J. Ding W. J. Fiatarone M. A. Evans W. J. Cannon J. G. (1993). Acute-phase response in exercise. III. Neutrophil and IL-1 beta accumulation in skeletal muscle. Am. J. Physiol. 265, R166–R172. 10.1152/ajpregu.1993.265.1.R166 8342683

[B23] Frost R. A. Nystrom G. J. Lang C. H. (2003). Lipopolysaccharide and proinflammatory cytokines stimulateinterleukin-6 expression in C2C12 myoblasts: Role of the jun NH2-terminal kinase. Am. J. Physiol. Regul. Integr. Comp. Physiol. 285, R1153–R1164. 10.1152/ajpregu.00164.2003 12842862

[B24] Frost R. A. Nystrom G. J. Lang C. H. (2002). Lipopolysaccharide regulates proinflammatory cytokine expression in mouse myoblasts and skeletal muscle. Am. J. Physiol. Regul. Integr. Comp. Physiol. 283, R698–R709. 10.1152/ajpregu.00039.2002 12185005

[B25] Fu X. Xiao J. Wei Y. Li S. Liu Y. Yin J. (2015). Combination of inflammation-related cytokines promotes long-term muscle stem cell expansion. Cell Res. 25, 655–673. 10.1038/cr.2015.58 25976405 PMC4456625

[B26] Garry D. J. Meeson A. Elterman J. Zhao Y. Yang P. Bassel-Duby R. (2000). Myogenic stem cell function is impaired in mice lacking the forkhead/winged helix protein MNF. Proc. Natl. Acad. Sci. U.S.A. 97, 5416–5421. 10.1073/pnas.100501197 10792059 PMC25843

[B27] Gayraud-Morel B. Le Bouteiller M. Commere P.-H. Cohen-Tannoudji M. Tajbakhsh S. (2018). Notchless defines a stage-specific requirement for ribosome biogenesis during lineage progression in adult skeletal myogenesis. Development 145, dev162636–12. 10.1242/dev.162636 30478226

[B28] Ge Y. Waldemer R. J. Nalluri R. Nuzzi P. D. Chen J. (2013). RNAi screen reveals potentially novel roles of cytokines in myoblast differentiation. PLoS One 8, e68068. 10.1371/journal.pone.0068068 23844157 PMC3699544

[B29] Gordon B. S. Kelleher A. R. Kimball S. R. (2013). Regulation of muscle protein synthesis and the effects of catabolic states. Int. J. Biochem. Cell Biol. 45, 2147–2157. 10.1016/j.biocel.2013.05.039 23769967 PMC3759561

[B30] Gordon J. R. Galli S. J. (1990). Mast cells as a source of both preformed and immunologically inducible TNF-alpha/cachectin. Nature 346, 274–276. 10.1038/346274a0 2374592

[B31] Grandvaux N. Servant M. J. ten Oever B. Sen G. C. Balachandran S. Barber G. N. (2002). Transcriptional profiling of interferon regulatory factor 3 target genes: Direct involvement in the regulation of interferon-stimulated genes. J. Virol. 76, 5532–5539. 10.1128/JVI.76.11.5532-5539.2002 11991981 PMC137057

[B32] Greenberg S. A. Pinkus J. L. Pinkus G. S. Burleson T. Sanoudou D. Tawil R. (2005). Interferon-alpha/beta-mediated innate immune mechanisms in dermatomyositis. Ann. Neurol. 57, 664–678. 10.1002/ana.20464 15852401

[B33] Griffin C. A. Apponi L. H. Long K. K. Pavlath G. K. (2010). Chemokine expression and control of muscle cell migration during myogenesis. J. Cell Sci. 123, 3052–3060. 10.1242/jcs.066241 20736301 PMC2931603

[B34] Haimes J. Kelley M. (2010). Demonstration of a ΔΔCq calculation method to compute relative gene expression from qPCR data. Thermo. Sci. Technol. Note, 1–4.

[B35] Hee J. S. Cresswell P. (2017). Viperin interaction with mitochondrial antiviral signaling protein (MAVS) limits viperin-mediated inhibition of the interferon response in macrophages. PLoS One 12, e0172236. 10.1371/journal.pone.0172236 28207838 PMC5313200

[B36] Hogan K. A. Cho D. S. Arneson P. C. Samani A. Palines P. Yang Y. (2018). Tumor-derived cytokines impair myogenesis and alter the skeletal muscle immune microenvironment. *Cytokine* **107** 107, 9–17. 10.1016/j.cyto.2017.11.006 PMC5916328 29153940

[B37] Howard E. E. Pasiakos S. M. Blesso C. N. Fussell M. A. Rodriquez N. R. (2020). Divergent roles of inflammation in skeletal muscle recovery from injury. Front. Physiology 11, 87–13. 10.3389/fphys.2020.00087 PMC7031348 32116792

[B38] Imaizumi T. Aizawa-Yashiro T. Watanabe S Matsumiya T. Yoshida H. Tatsuta T. (2013). TLR4 signaling induces retinoic acid-inducible gene-I and melanoma differentiation-associated gene 5 in mesangial cells. J. Nephrol. 26, 886–893. 10.5301/jn.5000254 23559071

[B39] Kelly-Scumpia K. M. Scumpia P. O. Delano M. J. Weinstein J. S. Cuenca A. G. Wynn J. L. (2010). Type I interferon signaling in hematopoietic cells is required for survival in mouse polymicrobial sepsis by regulating CXCL10. J. Exp. Med. 207, 319–326. 10.1084/jem.20091959 20071504 PMC2822595

[B40] Kim M.-J. Hwang S. Y. Imaizumi T. Yoo J. Y. (2008). Negative feedback regulation of RIG-I-mediated antiviral signaling by interferon-induced ISG15 conjugation. J. Virol. 82, 1474–1483. 10.1128/JVI.01650-07 18057259 PMC2224411

[B41] Kohno S. Ueji T. Abe T. Nakao R. Hirasaka K. Oarada M. (2011). Rantes secreted from macrophages disturbs skeletal muscle regeneration after cardiotoxin injection in Cbl-b-deficient mice. Muscle Nerve 43, 223–229. 10.1002/mus.21829 21254087

[B43] Kuru S. Inukai A. Kato T. Lian T. Kimura S. Sobue G. (2003). Expression of tumor necrosis factor-α in regenerating muscle fibers in inflammatory and non-inflammatory myopathies. Acta Neuropathol. 105, 217–224. 10.1007/s00401-002-0635-4 12557007

[B45] Langen R. C. Van Der Velden J. L. Schols A. M. Kelders M. C. Wouters E. F. Janssen-Heininger Y. M. (2004). Tumor necrosis factor-alpha inhibits myogenic differentiation through MyoD protein destabilization. FASEB J. 18, 227–237. 10.1096/fj.03-0251com 14769817

[B46] Lazure F. Blackburn D. M. Corchado A. H. Sahinyan K. Karam N. Sharanek A. (2020). Myf6/MRF4 is a myogenic niche regulator required for the maintenance of the muscle stem cell pool. EMBO Rep. 21, e49499. 10.15252/embr.201949499 33047485 PMC7726801

[B47] Li L.-F. Yu J. Zhang Y. Yang Q. Li Y. Zhang L. (2017). Interferon-Inducible oligoadenylate synthetase-like protein acts as an antiviral effector against classical swine fever virus via the MDA5-mediated type I interferon-signaling pathway. J. Virol. 91, e01514. 10.1128/JVI.01514-16 28331099 PMC5432864

[B48] Li N. Mu H. Zheng L. Li B. Wu C. Niu B. (2016). EIF2S3Y suppresses the pluripotency state and promotes the proliferation of mouse embryonic stem cells. Oncotarget 7, 11321–11331. 10.18632/oncotarget.7187 26863630 PMC4905476

[B49] Li Y. P. (2003). TNF-alpha is a mitogen in skeletal muscle. Am. J. Physiol. Cell Physiol. 285, C370–C376. 10.1152/ajpcell.00453.2002 12711593

[B50] Liu G. Q. Lee J.-H. Parker Z. M. Acharya D. Chiang J. J. van Gent M. (2021). ISG15-dependent activation of the sensor MDA5 is antagonized by the SARS-CoV-2 papain-like protease to evade host innate immunity. Nat. Microbiol. 6, 467–478. 10.1038/s41564-021-00884-1 33727702 PMC8103894

[B51] Liu S.-Y. Sanchez D. J. Aliyari R. Lu S. Cheng G. (2012). Systematic identification of type I and type II interferon-induced antiviral factors. Proc. Natl. Acad. Sci. U. S. A. 109, 4239–4244. 10.1073/pnas.1114981109 22371602 PMC3306696

[B52] Loo Y.-M. Gale M. (2011). Immune signaling by RIG-I-like receptors. Immunity 34, 680–692. 10.1016/j.immuni.2011.05.003 21616437 PMC3177755

[B53] Lu H. Huang D. Ransohoff R. M. Zhou L. (2011). Acute skeletal muscle injury: CCL2 expression by both monocytes and injured muscle is required for repair. FASEB J. 25, 3344–3355. 10.1096/fj.10-178939 21697550 PMC3177578

[B54] Lu Y.-C. Yeh W.-C. Ohashi P. S. (2008). LPS/TLR4 signal transduction pathway. Cytokine 42, 145–151. 10.1016/j.cyto.2008.01.006 18304834

[B55] MacMicking J. D. (2012). Interferon-inducible effector mechanisms in cell-autonomous immunity. Nat. Rev. Immunol. 12, 367–382. 10.1038/nri3210 22531325 PMC4150610

[B56] Majumdar G. Vera S. Elam M. B. Raghow R. (2015). A streamlined protocol for extracting RNA and genomic DNA from archived human blood and muscle. Anal. Biochem. 474, 25–27. 10.1016/j.ab.2014.12.021 25579785

[B57] Malathi K. Dong B. Gale M. Jr Silverman R. H. (2007). Small self-RNA generated by RNase L amplifies antiviral innate immunity. Nature 448, 816–819. 10.1038/nature06042 17653195 PMC3638316

[B58] Martinet C. Monnier P. Louault Y. Benard M. Gabory A. Dandolo L. (2016). H19 controls reactivation of the imprinted gene network during muscle regeneration. Development 143, 962–971. 10.1242/dev.131771 26980793

[B59] Martinez C. O. McHale M. J. Wells J. T. Ochoa O. Michalek J. E. McManus L. M. (2010). Regulation of skeletal muscle regeneration by CCR2-activating chemokines is directly related tomacrophage recruitment. Am. J. Physiol. Regul. Integr. Comp. Physiol. 299, R832–R842. –R842. 10.1152/ajpregu.00797.2009 20631294 PMC2944434

[B60] Martinon F. Mayor A. Tschopp J. (2009). The inflammasomes: Guardians of the body. Annu. Rev. Immunol. 27, 229–265. 10.1146/annurev.immunol.021908.132715 19302040

[B61] McKellar D. W. Walter L. D. Song L. T. Mantri M. Wang M. F. Z. De Vlaminck I. (2021). Large-scale integration of single-cell transcriptomic data captures transitional progenitor states in mouse skeletal muscle regeneration. Commun. Biol. 4, 1280. 10.1038/s42003-021-02810-x 34773081 PMC8589952

[B62] Mervis M. J. Matsumura M. S. Olsen Z. E. Hirschi-Budge K. M. Reynolds P. R. Arroyo J. A. (2020). The effect of gas6-axl double knockout on satellite cell proliferation and skeletal muscle regeneration after injury. FASEB J. 34, 1. 10.1096/fasebj.2020.34.s1.09814

[B63] Moneta G. M. Marafon D. P. Marasco E. Rosina S. Verardo M. Fiorillo C. (2019). Muscle expression of type I and type II interferons is increased in juvenile dermatomyositis and related to clinical and histologic features. Arthritis Rheumatol. 71, 1011–1021. 10.1002/art.40800 30552836

[B64] Munoz-Canoves P. Scheele C. Pedersen B. K. Serrano A. L. (2013). Interleukin-6 myokine signaling in skeletal muscle: A double-edged sword? FEBS J. 280, 4131–4148. 10.1111/febs.12338 23663276 PMC4163639

[B65] Nakano M. Fujii T. Hashimoto M. Yukawa N. Yoshifuji H. Ohmura K. (2012). Type I interferon induces CX3CL1 (fractalkine) and CCL5 (RANTES) production in human pulmonary vascular endothelial cells. Clin. Exp. Immunol. 170, 94–100. 10.1111/j.1365-2249.2012.04638.x 22943205 PMC3444721

[B66] Oprescu S. N. Yue F. Qiu J. Brito L. F. Kuang S. (2020). Temporal dynamics and heterogeneity of cell populations during skeletal muscle regeneration. iScience 23, 100993. 10.1016/j.isci.2020.100993 32248062 PMC7125354

[B67] Otani M. Furukawa S. Wakisaka S. Maeda T. (2020). A novel adipokine C1q/TNF-related protein 3 is expressed in developing skeletal muscle and controls myoblast proliferation and differentiation. Mol. Cell. Biochem. 409, 271–282. 10.1007/s11010-015-2531-y 26272338

[B68] Palade J. Pal A. Rawls A. Stabenfeldt S. Wilson-Rawls J. (2019). Molecular analysis of muscle progenitor cells on extracellular matrix coatings and hydrogels. Acta Biomater. 97, 296–309. 10.1016/j.actbio.2019.08.019 31415920

[B69] Richez C. Yasuda K. Watkins A. A. Akira S. Lafyatis R. Van Seventer J. (2010). TLR4 ligands induce IFN-alpha production by mouse conventional dendritic cells and human monocytes after IFN-beta priming. J. Immunol. 182, 820–828. 10.4049/jimmunol.182.2.820 PMC2858919 19124725

[B70] Rodgers J. T. King K. Y. Brett J. O. Cromie M. J. Charville G. W. Maguire K. K. (2014). mTORC1 controls the adaptive transition of quiescent stem cells from G0 to G(Alert). Nature 510, 393–396. 10.1038/nature13255 24870234 PMC4065227

[B71] Rodriguez-Outeiriño L. Hernandez-Torres F. Ramírez-de Acuña F. Matías-Valiente L. Sanchez-Fernandez C. Franco D. (2021). Muscle satellite cell heterogeneity: Does embryonic origin matter? Front. Cell Dev. Biol. 15 (9), 750534. 10.3389/fcell.2021.750534 PMC8554119 34722534

[B72] Salajegheh M. Kong S. W. Pinkus J. L. Walsh R. J. Liao A. Nazareno R. (2010). Interferon-stimulated gene 15 (ISG15) conjugates proteins in dermatomyositis muscle with perifascicular atrophy. Ann. Neurol. 67, 53–63. 10.1002/ana.21805 20186858 PMC2875060

[B73] Scaramozza A. Park D. Kollu S. Beerman I. Sun X. Rossi D. J. (2019). Lineage tracing reveals a subset of reserve muscle stem cells capable of clonal expansion under stress. Cell Stem Cell 24, 944–957.e5. 10.1016/j.stem.2019.03.020 31006621 PMC6597014

[B74] Sheehan S. M. Tatsumi R. Temm-Grove C. J. Allen R. E. (2000). HGF is an autocrine growth factor for skeletal muscle satellite cells *in vitro* . Muscle and Nerve 23, 239–245. 10.1002/(sici)1097-4598(200002)23:2<239:aid-mus15>3.0.co;2-u 10639617

[B75] Stok J. E. Quiroz M. E. V. van der Veen A. G. (2020). Self RNA sensing by RIG-I-like receptors in viral infection and sterile inflammation. *J. Immunol* **205** 205, 883–891. 10.4049/jimmunol.2000488 32769143

[B76] Strömberg A. Olsson K. Dijksterhuis J. P. Rullman E. Schulte G. Gustafsson T. (2016). CX3CL1--a macrophage chemoattractant induced by a single bout of exercise in human skeletal muscle. Am. J. Physiol. Regul. Integr. Comp. Physiol. 310, R297–R304. 10.1152/ajpregu.00236.2015 26632602

[B77] Suárez-Calvet X. Gallardo E. Nogales-Gadea G. Querol L. Navas M. Diaz-Manera J. (2014). Altered RIG-I/DDX58-mediated innate immunity in dermatomyositis. J. Pathol. 233, 258–268. 10.1002/path.4346 24604766

[B78] Teixeira C. F. Zamuner S. R. Zuliani J. P. Fernandes C. M. Cruz-Hofling M. A. Fernandes I. (2003). Neutrophils do not contribute to local tissue damage, but play a key role in skeletal muscle regeneration, in mice injected with *Bothrops asper* snake venom. Muscle Nerve 28, 449–459. 10.1002/mus.10453 14506717

[B79] Tidball J. G. (2017). Regulation of muscle growth and regeneration by the immune system. Nat. Rev. Immunol. 17 (3), 165–178. 10.1038/nri.2016.150 28163303 PMC5452982

[B80] Torrente Y. El Fahime E. Caron N. J. Bo R. D. Belicchi M. Pisati F. (2003). Tumor necrosis factor-alpha (TNF-alpha) stimulates chemotactic response in mouse myogenic cells. Cell Transpl. 12, 91–100. 10.3727/000000003783985115 12693669

[B81] Venereau E. Casalgrandi M. Schiraldi M. Antoine D. J. Cattaneo A. De Marchis F. (2012). Mutually exclusive redox forms of HMGB1 promote cell recruitment or proinflammatory cytokine release. J. Exp. Med. 209, 1519–1528. 10.1084/jem.20120189 22869893 PMC3428943

[B82] Wang P. Han X. Mo B. Huang G. Wang C. (2017). LPS enhances TLR4 expression and IFN-γ production via the TLR4/IRAK/NF-κB signaling pathway in rat pulmonary arterial smooth muscle cells. Mol. Med. Rep. 16, 3111–3116. 10.3892/mmr.2017.6983 28714001 PMC5547977

[B83] Warren G. L. Hulderman T. Jensen N. McKinstry M. Mishra M. Luster M. I. (2002). Physiological role of tumor necrosis factor-α in traumatic muscle injury. FASEB J. 16, 1630–1632. 10.1096/fj.02-0187fje 12207010

[B84] Yahiaoui L. Gvozdic D. Danialou G. Mack M. Petrof B. J. (2008). CC family chemokines directly regulate myoblast responses to skeletal muscle injury. J. Physiol. 586, 3991–4004. 10.1113/jphysiol.2008.152090 18566004 PMC2538927

[B85] Yang J. Zhao Y. Shao F. (2015). Non-canonical activation of inflammatory caspases by cytosolic LPS in innate immunity. Curr. Opin. Immunol. 32, 78–83. 10.1016/j.coi.2015.01.007 25621708

[B86] Yang W. Hu P. (2018). Skeletal muscle regeneration is modulated by inflammation. J. Orthop. Transl. 13, 25–32. 10.1016/j.jot.2018.01.002 PMC5892385 29662788

[B87] Yin H. Price F. Rudnicki M. A. (2013). Satellite cells and the muscle stem cell niche. Physiol. Rev. 93, 23–67. 10.1152/physrev.00043.2011 23303905 PMC4073943

[B88] Zhang L. Ran L. Garcia G. E. Wang X. H. Han S. Du J. (2009). Chemokine CXCL16 regulates neutrophil and macrophage infiltration into injured muscle, promoting muscle regeneration. Am. J. Pathol. 175, 2518–2527. 10.2353/ajpath.2009.090275 19893053 PMC2789607

[B90] Zhu H. Xiao F. Wang G. Wei X. Jiang L. Chen Y. (2016). STAT3 regulates self-renewal of adult muscle satellite cells during injury-induced muscle regeneration. Cell Rep. 16, 2102–2115. 10.1016/j.celrep.2016.07.041 27524611

