# Supplementary material for: Single cell analysis reveals satellite cell heterogeneity for proinflammatory chemokine expression
Source: Front Cell Dev Biol. 2023 Mar 27;11:1084068. doi: 10.3389/fcell.2023.1084068 (PMC10083252; doi:10.3389/fcell.2023.1084068)
Supplement: Supplementary file 3 [file Table1.DOCX]

| **Gene** | **Forward Primer** | **Reverse Primer** |
| --- | --- | --- |
| Ccl2 | 5’-GAGAGCTACAAGAGGATCACC-3’ | 5’-GAATTTGCCGTGAGTGGAGT-3’ |
| Ccl5 | 5’-AGATCTCTGCAGCTGCCTCA-3’ | 5’-GGAGCATTGCTGCTGGTGTAG-3’ |
| Ccl7 | 5’-CAGAAGGATCACCAGTAGTCGG-3’ | 5’-ATAGCCTCCTCGACCCACTTCT-3’ |
| Cxcl1 | 5’-TCCAGAGCTTGAAGGTGTTGCC-3’ | 5’-AACCAAGGGAGCTTCAGGGTCA-3’ |
| Cxcl10 | 5’-ATCATCCCTGCGAGCCTATCCT-3’ | 5’-GACCTTTTTTGGCTAAACGCTTTC-3’ |
| Gapdh | 5’CCGCATCTTCTTGTGCAGT-3’ | 5’-GAATTTGCCGTGAGTGGAGT-3’ |
| Ifih-1 | 5’-TGCGGAAGTTGGAGTCAAAGCG-3’ | 5’-CACCGTCGTAGCGATAAGCAGA-3’ |
| IL-6 | 5’-TACCACTTCACAAGTCGGAGGC-3’ | 5’-CTGCAAGTGCATCATCGTTGTTC-3’ |
| ISG15 | 5’-CATCCTGGTGAGGAACGAAAGG-3’ | 5’-CTCAGCCAGAACTGGTCTTCGT-3’ |
| TNFa | 5’-GGTGCCTATGTCTCAGCCTCTT-3’ | 5’-GCCATAGAACTGATGAGAGGGAG-3’ |
