# Supplementary material for: Single cell analysis reveals satellite cell heterogeneity for proinflammatory chemokine expression
Source: Front Cell Dev Biol. 2023 Mar 27;11:1084068. doi: 10.3389/fcell.2023.1084068 (PMC10083252; doi:10.3389/fcell.2023.1084068)
Supplement: Supplementary file 5 [file Table5.DOCX]

**SUPPLEMENTAL LEGENDS**

**Supplemental Table 1: Primer sequences for RT-qPCR.**

**Supplemental Table 2: Differentially expressed genes from PBS-treated MuSC clusters as generated by Seurat R package.**

**Supplemental Table 3: Differentially expressed genes from LPS-treated MuSC clusters as generated by Seurat R package.**

**Supplemental Table 4: RT-qPCR of LPS-induced transcription of chemokines in primary MuSC cultures**
