# Supplementary material for: Single cell analysis reveals satellite cell heterogeneity for proinflammatory chemokine expression
Source: Front Cell Dev Biol. 2023 Mar 27;11:1084068. doi: 10.3389/fcell.2023.1084068 (PMC10083252; doi:10.3389/fcell.2023.1084068)
Supplement: Supplementary file 6 [file Table4.DOCX]

| **Chemokine** | **Fold Change** | **Standard Deviation** |
| --- | --- | --- |
| Cxcl1 | 87.97 | 2.50 |
| Ccl2 | 478.77 | 1.50 |
| Ccl5 | 11.98 | 0.70 |
| Ccl7 | 83.26 | 0.64 |
| Cxcl10 | 60.34 | 0.57 |
